# Wastewater Treatment for Carbon Dioxide Removal

**DOI:** 10.1021/acsomega.3c04231

**Published:** 2023-10-19

**Authors:** Vhahangwele Masindi, Spyros Foteinis, Phil Renforth, Efthalia Chatzisymeon

**Affiliations:** † Magalies Water, Scientific Services, Research & Development Division, Erf 3475, Stoffberg street, Brits 0250, South Africa; ‡ Department of Environmental Sciences, College of Agriculture and Environmental Sciences, University of South Africa (UNISA), P.O. Box 392, Florida 1710, South Africa; § Research Centre for Carbon Solutions, School of Engineering and Physical Sciences, Heriot-Watt University, Edinburgh EH14 4AS, United Kingdom; ∥ School of Engineering, Institute for Infrastructure and Environment, University of Edinburgh, Edinburgh EH9 3JL, United Kingdom

## Abstract

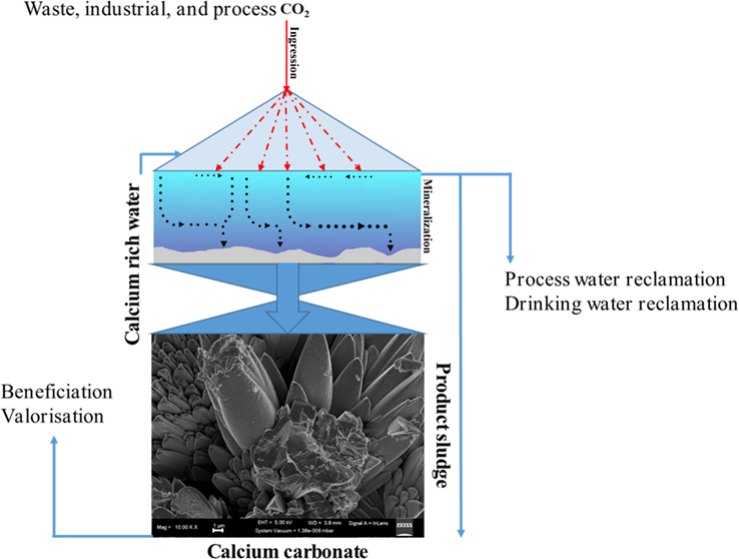

Wastewater treatment is notorious for its hefty carbon footprint, accounting for 1–2% of global greenhouse gas (GHG) emissions. Nonetheless, the treatment process itself could also present an innovative carbon dioxide removal (CDR) approach. Here, the calcium (Ca)-rich effluent of a phosphorus (P) recovery system from municipal wastewater (P recovered as calcium phosphate) was used for CDR. The effluent was bubbled with concentrated CO_2_, leading to its mineralization, i.e., CO_2_ stored as stable carbonate minerals. The chemical and microstructural properties of the newly formed minerals were ascertained by using state-of-the-art analytical techniques. FTIR identified CO_3_ bonds and carbonate stretching, XRF and SEM-EDX measured a high Ca concentration, and SEM imaging showed that Ca is well distributed, suggesting homogeneous formation. Furthermore, FIB-SEM revealed rhombohedral and needle-like structures and TEM revealed rod-like structures, indicating that calcium carbonate (CaCO_3_) was formed, while XRD suggested that this material mainly comprises aragonite and calcite. Results imply that high-quality CaCO_3_ was synthesized, which could be stored or valorized, while if atmospheric air is used for bubbling, a partial direct air capture (DAC) system could be achieved. The quality of the bubbled effluent was also improved, thus creating water reclamation and circular economy opportunities. Results are indicative of other alkaline Ca-rich wastewaters such as effluents or leachates from legacy iron and steel wastes (steel slags) that can possibly be used for CDR. Overall, it was identified that wastewater can be used for carbon mineralization and can greatly reduce the carbon footprint of the treatment process, thus establishing sustainable paradigms for the introduction of CDR in this sector.

## 1 Introduction

Climate change is a growing issue of emerging concern, with its impacts spanning from weather extremes to rising sea levels, loss of habitats and biodiversity, and loss of life. Climate change also affects wastewater treatment systems. For example, weather extremes can lead to the release of untreated wastewater, while at the same time the treatment process itself, which is energy intensive, is responsible for direct and indirect greenhouse gas (GHG) emissions (e.g., CO_2_, N_2_O, and CH_4_).^1^ Specifically, ∼3% of the global electricity consumption is directed to wastewater treatment^2^ and contributes between 1 and 2% of the global GHG emissions.^3^ However, wastewater treatment is an essential perquisite for addressing water pollution and safeguarding human health and the environment.^1,4^

As such, the industry is under pressure to achieve carbon neutrality, with energy savings and resource recovery opportunities for producing carbon-based materials being encouraged.^3^ A pathway that has received little attention is the use of wastewater for carbon dioxide removal (CDR),^2^ thus offsetting the environmental footprint of the treatment process (e.g., through energy, chemicals, or building material production)^5^ and possibly even lead to carbon negative systems. Wastewater treatment-based CDR can include microalgae bioremediation,^6^ CO_2_ mineralization in municipal wastewater (MWW) by using the UV/H_2_O_2_ process and an ion-exchange membrane,^7^ CO_2_ neutralization with Zn^2+^ precipitation in tannery unhairing wastewater treatment,^8^ and the release of alkalinity-containing wastewater for ocean alkalinity enhancement (OAE).^9,10^ The latter can also counteract the effect of treated wastewater on the carbonate chemistry of the oceans, which exacerbates coastal water acidification.^11^

Here, a novel approach for wastewater treatment-based CDR is examined, whereby a calcium (Ca)-rich wastewater effluent is used for CO_2_ uptake. Specifically, the depletion of natural phosphate rock reserves has resulted in increasing efforts to recover the phosphorus (P) contained in MWW.^12^ This can be mainly achieved through P precipitation/crystallization and toward the synthesis and precipitation of either calcium phosphate or magnesium ammonium phosphate (MAP, also known as struvite).^13^ In both cases, alkalinity, as a calcium (Ca) or magnesium (Mg) oxide/hydroxide, respectively, is added to MWW to promote P precipitation/crystallization. This results in a P-depleted effluent that is enriched in Ca and/or Mg and is alkaline in nature. For example, pH values of 11.5^14,15^ and 10.5^16^ have been reported for effluents from Ca (calcium phosphate)- and Mg (struvite)-based P-recovery systems from real MWW. As such, the pH of these effluents is above the universal standard for wastewater discharge compliance (pH values in the range 6 to 9), requiring correction, while the high Ca and/or Mg values render these effluents promising candidates for carbon mineralization. Therefore, the feasibility of using such effluents for CDR (CO_2_ mineralization) was examined for the first time using effluents emanating from the calcium phosphate recovery system from MWW.

## 2 Materials and Methods

### 2.1 Sample Collection and Chemical Reagent Procurement

Calcium phosphate can be synthesized from MWW through calcium hydroxide (Ca(OH)_2_) addition, also generating a Ca-rich alkaline effluent,^17^ described herein as calcium phosphate wastewater (CPW). Here, the P content of MWW was fully removed, with pH values reaching as high as 12.5 before stabilizing at 11.5 after treatment. As such, the Mg content from MWW was also removed. However, due to its high pH (>9), electrical conductivity (EC), and Ca values, among others, this effluent is unfit for release to the environment. Therefore, the feasibility of using this effluent for CO_2_ mineralization, i.e., reacting its Ca content with CO_2_ toward carbonate mineral formation and pH correction, was examined. In doing so, CPW would also be treated (Ca and other minor impurities would be removed and hardness and EC would reduce), creating opportunities for water reclamation. The newly formed carbonate minerals could also be valorized, e.g., used as fillers in the plastic industry^18^ or simply stored. The CPW was collected in 25 L high-density polyethylene (HDPE) containers, while to remove suspended solids (debris were not present), it was first passed through a perforated filter paper. It was then stored in a dark and cool place until use for the CO_2_ mineralization experimental studies.

CO_2_ contained in air can equilibrate passively with CPW. However, this could be a slow process. Reaction rates can be enhanced by bubbling air or catalyzed when using concentrated/pure CO_2_. The latter is often used for correcting the pH of wastewater effluents.^19^ It has also been used to examine the direct carbonation of aqueous flue gas desulfurization gypsum,^20^ and it can be the byproduct of industrial activities (e.g., flue gas from oxyfuel combustion typically contains more than 95% CO_2_).^21^ Furthermore, the output of direct air capture (DAC) that is intended for geological storage contains typically over 99% CO_2_ and it is highly likely that this CDR technology would be colocated with DAC. For this reason, here, pure CO_2_ was considered for the direct carbonation of CPW. To this end, industrial grade CO_2_ was procured from African Oxygen (Afrox) Pty (Ltd.), South Africa and used for the mineralization experiments. The CO_2_ was bubbled directly from the gas cylinder. In scaled up systems, the concentrated CO_2_ can be provided through aDAC system or point sources (e.g., flue gases). However, in the latter case, emission reductions instead of removals would be achieved. It may be possible to design passive contact systems (baffles, cascades, and reed beds) for gas exchange, similar to those used for oxygenation of acid mine waters (although we do not explore these here).

### 2.2 Characterization Techniques

#### 2.2.1 Aqueous Samples Characterization

The main parameters of the aqueous samples, i.e., MWW, CPW, and CO_2_-bubbled effluent, were measured at the ISO/IEC 17025:2017 accredited laboratory (Magalies Water Services, Brits, North West, South Africa). Specifically, the pH, temperature, and EC were measured using an HQ40d Portable Meter (Hach Company). The DR6000 spectrophotometer (Hach Company) was used to measure COD, orthophosphate, nitrate, and ammonia in MWW (highly concentrated sample), and the Gallery Plus Discrete Analyzer (Thermo Fisher Scientific) was used to measure the same parameters in the produced effluents (less concentrated samples). Metals in these effluents were measured by inductively coupled plasma optical emission spectrometry (ICP-OES) (Agilent 5110 ICP-OES using the Vertical Dual View configuration and the SPS 4 Auto sampler). To assess biological contamination, the total plate count (TPC), the total coliforms, and*Escherichia coli* (*E. coli*), were measured; the latter two were measured using the U.S. EPA-approved Colilert test (Idexx Laboratories).

#### 2.2.2 Solid Samples Characterization

To verify the fate of the captured CO_2_, the newly synthesized carbonate minerals were characterized. Specifically, the mineralogical properties were ascertained using X-ray diffraction (XRD) (Philips PW 1710 equipped with a graphite secondary monochromatic source), and the elemental composition was ascertained using X-ray fluorescence (XRF) (Thermo Scientific ARL 9400 coupled with Win-XRF software). For context, the elemental composition of commercially available calcium carbonate was also examined. The morphological and elemental properties were ascertained using a high-resolution field emission scanning electron microscope (FE-SEM) (Carl Zeiss AURIGA crossbeam workstation using SmartSEM software) coupled with focused ion beam (FIB) and energy-dispersive X-ray spectroscopy (EDX). SEM-EDX was used to obtain the (surface) elemental composition, whereas FIB-SEM was used to capture ultrahigh-resolution images at the micro- and nanometer levels. Furthermore, a Fourier transform infrared (FTIR) spectrometer (PerkinElmer Spectrum 100 fitted with the attenuated total reflectance (ATR) sampling accessory) was used to identify the functional groups, whereas the structural characteristics at the nanoscale level were further ascertained using high-resolution transmission electron microscopy (TEM) (JEOL TEM-2100 electron microscope), equipped with EDX. Finally, the National Institute of Standards and Technology (NIST) standards were used for quality control and calibration of the instruments, while all analyses of the solid samples were performed in an ISO/IEC 17025:2017 accredited laboratory at the Council for Scientific and Industrial Research (CSIR), Pretoria, South Africa.

### 2.3 Experimental Setup

All experiments were performed at bench scale, whereby CPW was bubbled with pure CO_2_ toward carbonate mineral synthesis and precipitation. To examine the effect of the CO_2_ reaction time with CPW, different CO_2_ bubbling durations were considered, i.e., 2.5, 5, 10, 15, 20, 25, 30, 45, 60, and 90 min. Then, the CO_2_-bubbled effluent was left to settle and the produced sludge was collected and characterized. A conceptual illustration of the overall system, including the recovery of P from MWW using Ca(OH)_2_ and possible water recovery opportunities, is shown in Figure S1. Therefore, with the overall process, both circular economy and CRD could be introduced in wastewater treatment.

## 3 Results and Discussion

### 3.1 Effect of Bubbling Duration on the pH, Electrical Conductivity, and Calcium Concentration

**Figure 1:**
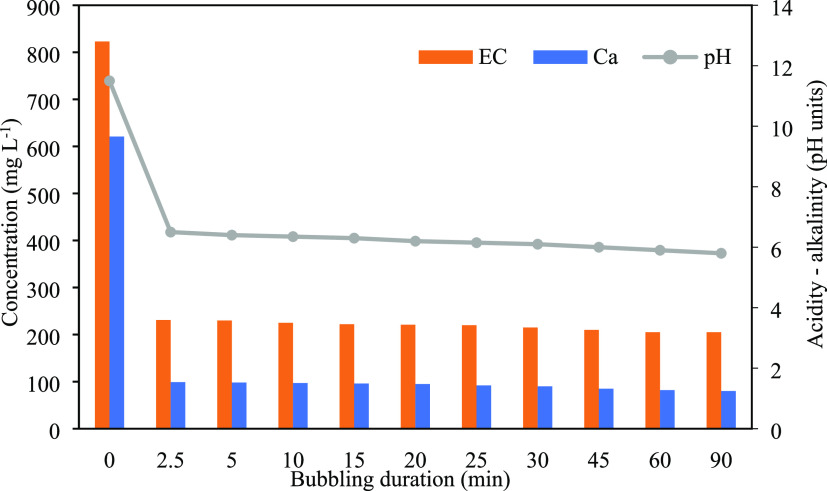
Effect of the CO_2_ bubbling duration on the pH, EC, and Ca levels of the bubbled effluent. Conditions: dosing CO_2_ directly from the cylinder, ambient pH, and room temperature.

The effect of the CO_2_ contact time on the pH, EC, and Ca levels of the CPW was examined by using the ten aforementioned bubbling durations. The results are summarized in Figure 1, where a rapid decrease is observed across the examined parameters from the early start of the examined contact times, i.e., from the 2.5 min bubbling duration. Specifically, the initial pH value of the CPW effluent was 11.5, and this was corrected to around 6.5, i.e., within the universal standard for wastewater discharge, in the first examined CO_2_ bubbling duration. Thereafter, the pH only slightly reduces with increasing bubbling duration. This is also the case for EC and Ca, both of which rapidly decreased after 2.5 min of bubbling (∼72%, from 823 to 231 mS cm^–1^, and ∼84%, from 621 to 99 mg L^–1^, respectively). Thereafter, both EC and Ca only slightly reduce with increasing bubbling duration, suggesting that their percentage removals have reached a plateau. Results suggest that a fast reaction or concurrent reactions between CO_2_ and CPW took place in the first few minutes of their interaction and thereafter the reaction(s) kinetics appear to have drastically reduced. This implies that when using pure CO_2_, only a few minutes of bubbling suffices to remove dissolved solids, mainly Ca, therefore reducing EC and also correcting the pH through acidity addition.

### 3.2 Quality of the Bubbled Effluent

To provide insight into the quality of the bubbled effluent, its physicochemical and microbial characteristics were further examined for the 2.5 min bubbling duration. For context, the quality of the raw MWW and CPW was also examined. As shown in Table 1, MWW comprised elevated levels of microbial contaminants (*E. coli*, total coliforms, and TPC), along with other contaminants, such as phosphate and ammonia, which are typically encountered in MWW. On the other hand, CPW contained increased levels of pH (from 7.3 in MWW to 11.5 in CPW), EC (from 97 to 823 mS cm^–1^), and Ca (from 21 to 621 mg L^–1^). Complete deactivation of microbial contaminants was also observed, which reduces the need for heated unpressurized CO_2_ bubbling^22^ or supercritical CO_2_ microbubbles^23^ for their deactivation when using CO_2_ bubbling. Furthermore, P and Mg were practically removed, while ammonia and chemical oxygen demand (COD) concetrations were also reduced (∼72 and ∼29%, respectively). Finally, the pH of the CO_2_-bubbled effluent was corrected (pH 6.5), and Ca and EC were greatly reduced (∼84 and ∼72%, respectively). As was expected, biological contamination was not identified in the bubbled effluent, whereas compared to CPW the remaining examined parameters reduced to a greater (e.g., ∼43% for sulfate) or lesser (e.g., ∼8% for ammonia and ∼1% for COD) extent.

**Table 1:** Physicochemical and Microbial Properties of Municipal Wastewater (MWW), Ca-Rich Alkaline Effluent (CPW), and Bubbled Effluent (CPW Following the Reaction with CO_2_ When a 2.5 min Bubbling Duration is Considered)

parameters	units	MWW	CPW	CO_2_ bubbled
E. coli (Colilert test)	MPN 100 mL^–1^	2420	ND^a^	ND
Total coliforms (Colilert test)	MPN 100 mL^–1^	24200	ND	ND
Total plate count (TPC)	count 1 mL^–1^	684000	<30	<30
pH @ 25 °C	-	7.3	11.5	6.5
Electrical conductivity (EC) at 25 °C	mS m^–1^	97	823	231
Sulfate	mg L^–1^ SO_4_	56	30	17
Ammonia	mg L^–1^	112.1	31.1	28.7
Dissolved sodium	mg L^–1^ Na	31	30	25.7
Dissolved zinc	mg L^–1^ Zn	<0.02	<0.02	<0.02
Dissolved iron	μg L^–1^ Fe	<0.37	<0.37	<0.37
Dissolved manganese	μg L^–1^ Mn	<0.09	<0.09	<0.09
Orthophosphate	mg L^–1^ P	79.5	<0.03	<0.03
Chemical oxygen demand (COD)	mg L^–1^	393	278	275
Dissolved calcium	mg L^–1^ Ca	21	621	99
Dissolved magnesium	mg L^–1^ Mg	25	0.03	0.01
Dissolved potassium	mg L^–1^ K	73	69	65

a ND = non-detected, i.e., below the detection limit.

Therefore, results suggest that CO_2_ bubbling improved CPW’s quality, with the main parameters of concern in the bubbled effluent being ammonia and COD, but their levels were not particularly high. Therefore, aeration (e.g., using existing aeration tanks/basins) might suffice to remove/strip ammonia and reduce COD and therefore meet the wastewater discharge standards. Water reclamation opportunities might also be available, but these will require a higher degree of treatment. For example, aeration and/or coagulation–flocculation (using readily available coagulants and flocculants) could be used to reclaim irrigation or industrial water or even produce water for aquifer recharge. Drinking water might also be reclaimed, but this will require even more robust treatment such as a combination of coagulation–flocculation and reverse osmosis (RO). As such, apart from CDR, zero liquid discharge (ZLD) and circular economy paradigms could also be introduced in wastewater treatment.

### 3.3 Analyses of the Recovered Solid Material

#### 3.3.1 X-ray Fluorescence

The elemental composition of the recovered material (carbonate minerals) was estimated using XRF. For context, the elemental composition of commercially available calcium carbonate (CaCO_3_) was also estimated since, most likely, the interaction of CO_2_ with the Ca content of CPW will lead to CaCO_3_ formation. Results are shown in Table S1, and as was expected, Ca was the main constituent in both matrices, while traces of other elements were also identified, particularly in the recovered material. Specifically, the commercial CaCO_3_ mostly comprised CaO (94.75%), followed to a much lesser extent by MgO (0.52%) and Na_2_O (0.23%) and other traces which are typical impurities contained in ores of calcium such as limestone.^24^ On the other hand, the recovered material mainly comprised CaO (98.45%), along with other impurities such as Na_2_O (0.43%), SrO (0.28%), SO_4_ (0.15%), and MgO (0.10%), which were presented in MWW and/or in the matrix of Ca(OH)_2_ which was used to recover P from MWW. Therefore, results suggest that the interaction of CO_2_ with CPW leads to CO_2_ mineralization. As expected, the main mineral that was synthesized was CaCO_3_, while other carbonate minerals might also be formed, such as magnesium and sodium carbonate, which could further improve the CDR potential. When only accounting for the Ca that has been removed from CPW (Table 1) in the form of stable CaCO_3_, it is inferred that more than 0.5 kg of CO_2_, in the form of CaCO_3_, can be removed per m^3^ of the bubbled effluent. Finally, the very high concentration of Ca in its matrix suggests that the synthesized CaCO_3_ is of high purity and could possibly be used in industrial applications.

#### 3.3.2 Energy-Dispersive X-ray Spectroscopy

**Figure 2:**
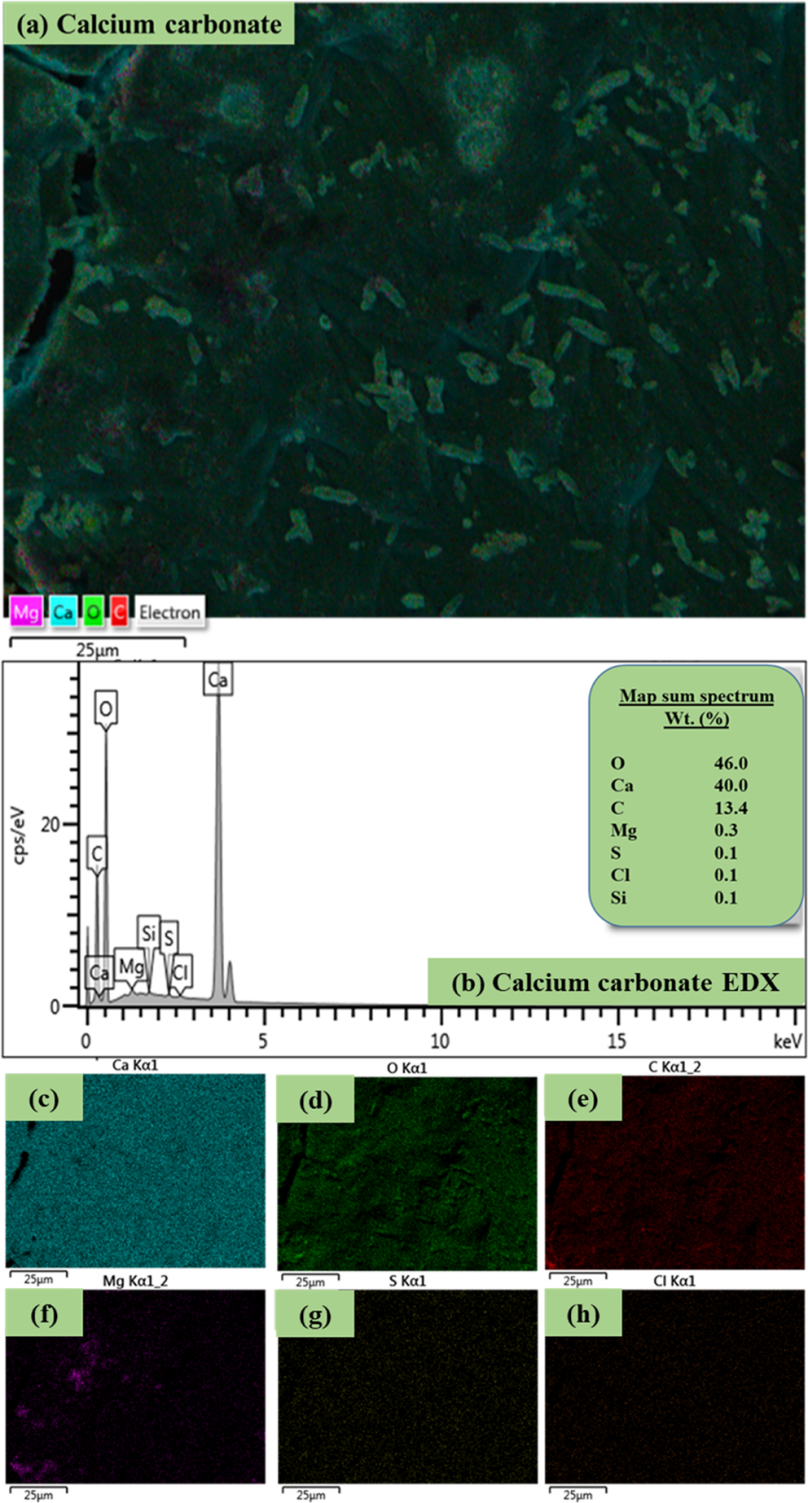
The (a) SEM electron image, (b) the EDX map sum spectrum, and the EDX layered images of (c) Ca, (d) O, (e) C, (f) Mg, (g) S, and (h) Cl.

To gain insight into the spatial distribution of Ca in the synthesized CaCO_3_ and its surface elemental composition, SEM-EDX was employed. Results are shown in Figure 2. The SEM electron image revealed that Ca is well distributed in the synthesized CaCO_3_ matrix (Figure 2a). Needle-like structures were dispersed across its surface and this morphology is consistent with aragonite, where needle-like particles of ∼20 μm length (aspect ratio 8–12) have been reported.^25^

Regarding the surface composition of the synthesized CaCO_3_, the EDX sum spectrum (the average that is calculated from all spectral imaging data acquired from all of the pixels in the electron image) identified O (46%), Ca (40%), and C (13.4%) as the main elements, along with traces of Cl, Mg, Si, and S (Figure 2b). It should be noted that due to the limitations of EDX analysis, C and O can be reliably identified but not accurately quantified. Here, to provide some insight on the concentrations of these two elements, carbon coating was not used during the EDX measurements, while their measured values could allude that, on a molar mass basis, the C and O concentration of these elements is similar to that of CaCO_3_. The map sum spectrum results also suggested that the produced CaCO_3_ is of high purity. Finally, the EDX elemental mapping identified the spectral features (intensity color) associated with Ca (Figure 2c), O (Figure 2d), C (Figure 2e), Mg (Figure 2f), S (Figure 2g), and Cl (Figure 2h). As was expected, the computed colorized layer of Ca had a higher intensity, followed by O and C, while Mg, S, and Cl gave very low intensities (dark colors). This is in agreement with the EDX map sum spectrum (Figure 2b) and the XRF results (section 3.3.2).

#### 3.3.3 Mineralogy Composition

The mineralogical characteristics of the synthesized CaCO_3_, along with commercially available CaCO_3_, were examined using XRD and the results are shown in Figure S2. It was identified that both materials comprise aragonite and calcite, but at different concentrations. Specifically, aragonite was observed to be between 30 and 85 2-theta (2 θ) degrees, while calcite was observed between 25 and 70 2-θ°. The high crystallinity in the diffractogram denotes that the synthesized CaCO_3_ is a crystalline mineral. Furthermore, there is a clear alignment between the 2-theta degrees of the synthesized and commercially available CaCO_3_, hence confirming that the synthesized material could be valorized, e.g., used for industrial applications. However, a clear difference on the weight percentages (wt %) of the measured minerals was also identified. Specifically, commercial CaCO_3_ comprised ∼98% calcite and ∼2% aragonite, whereas the synthesized CaCO_3_ comprised ∼25% calcite and ∼75% aragonite. It should be noted that the formation of anhydrous crystalline polymorphs of CaCO_3_ is greatly affected by the pH of the solution, degree of saturation, temperature, pressure, reaction time, impurities, and other parameters.^26^ Here, aragonite formation could be promoted by impurities contained in CPW, such as Mg, which can favor aragonite formation.^27^ The pH of the solution could also be a contributing factor, since pH values higher than 11 favor calcite formation, while aragonite is preferentially formed at pH 9 to 11.^26^

### 3.4 Focused Ion Beam Scanning Electron Microscopy

**Figure 3:**
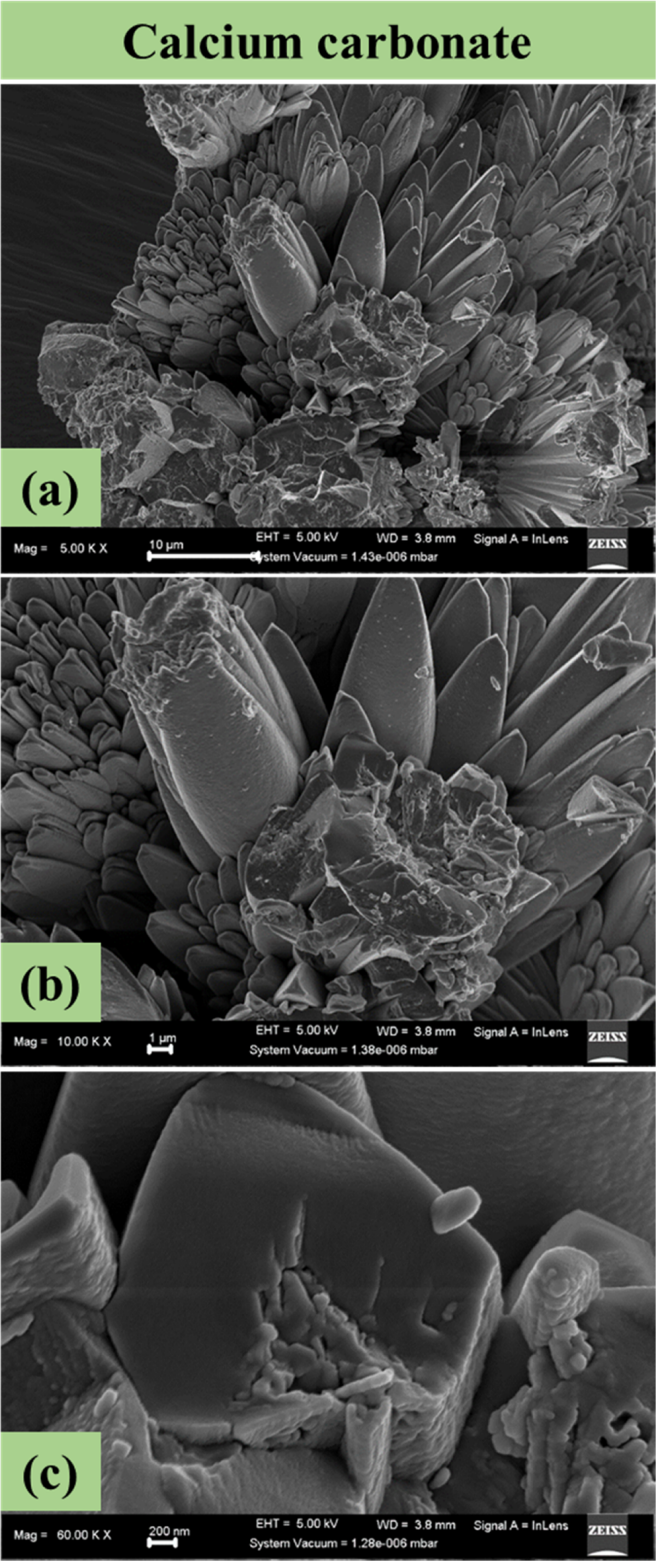
High-resolution FIB-SEM images showing the morphological properties of the synthesized CaCO_3_ at (a) 10 μm, (b) 1 μm, and (c) 200 nm magnification.

The morphological and microstructural properties of the synthesized CaCO_3_ were identified using FIB-SEM. High-resolution images were obtained at different magnifications, which highlight that the synthesized CaCO_3_ is homogeneous in nature and mainly comprises needle- and flower-like structures stemmed from the same origin (Figure 3). These structures represent the presence of calcite, which has a rhombohedral shape, and aragonite, which has a rod- or needle-like particle shape.^28^ Under normal conditions, the most thermodynamically stable form of CaCO_3_ is calcite (β-CaCO_3_), while, as mentioned above, other polymorphs of CaCO_3_ such as aragonite (λ-CaCO3) and vaterite (μ-CaCO3) can be formed at certain pH and temperature conditions.^29^ It should be noted that vaterite has a spherical shape,^28^ and therefore it was not identified herein. Other impurities contained in CPW, such as Mg, could also contribute to the formation of the observed structures, e.g., magnesium calcite also has a rhombohedral shape.^30^ Overall, from the FIB-SEM images, calcite and aragonite phases can be clearly distinguished in the synthesized CaCO_3_. Finally, the microstructural and morphological properties were observed to remain similar regardless of the employed magnification (ranging from 10 μm (Figure 3a) to 1 μm (Figure 3b) and 200 nm (Figure 3c)), hence suggesting uniformity and homogeneity of this material. The distinctive and fully crystallized nature further highlights the homogeneity.

### 3.5 Fourier Transform Infrared Spectroscopy

The metal functional groups of the synthesized and commercially available CaCO_3_ were identified using FTIR and the results are shown in Figure S3. Specifically, both matrices were found to include hydroxyl and carbonate bonds. Interestingly, similar stretching was observed in both materials and at the same wavenumber. This denotes that the synthesized material is of high purity, as is the case with the commercially available material. The results are typical for a CaCO_3_-based material. For example, the peaks at 707 and 873 cm^–1^ correspond to the in-plane and out-plane bending, respectively, and the peak at 1418 cm^–1^ to asymmetrical stretching of O–C–O.^31^ Similar results have been reported for these adsorption bands,^32,33^ while the low peaks near the 3000 cm^–1^ correspond to the broad −OH band. Therefore, the presence of carbonate denotes the presence of a carbonate mineral, i.e., CaCO_3_, whereas the presence of a hydroxyl group suggests that both synthetic and commercial CaCO_3_ can also include some (based on the transmittance data peaks) water or most likely hydrates in their matrices.

### 3.6 High-Resolution Transmission Electron Microscopy

**Figure 4:**
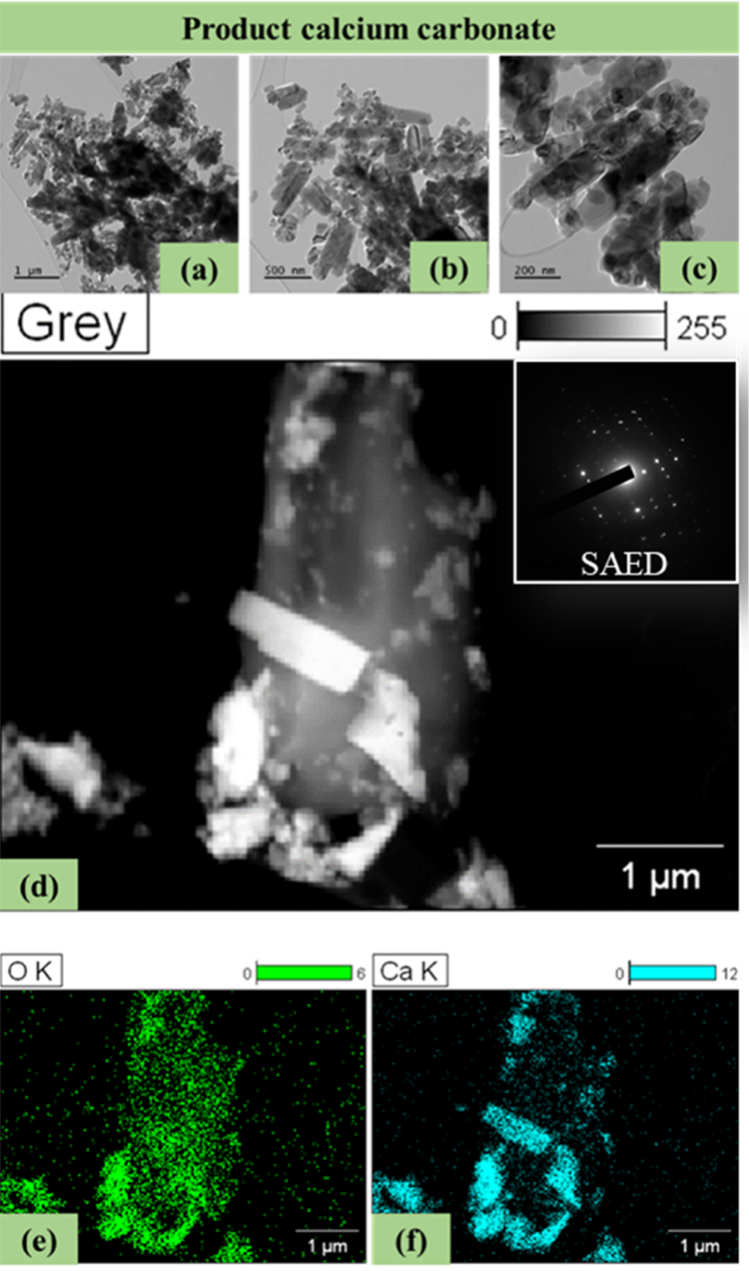
The HR-TEM micrographs showing the morphological properties of the synthesized CaCO_3_ at different magnifications: (a) 1 μm, (b) 500 nm, and (c) 200 nm; the (d) SAED diffraction pattern; and the map sum spectrum of (e) O and (f) Ca.

The micrographs of the synthesized CaCO_3_ were obtained using HR-TEM and the results are shown in Figure 4. The micrographs, at different magnifications, i.e., 1 μm (Figure 4a), 500 nm (Figure 4b), and 200 nm (Figure 4c), clearly show that this material comprises rod-like particles, overlapping on top of each other. Based on these results, it appears that nanocrystals of different sizes have been formed, with sizes in the nanometer (nm) scale. In general, CaCO_3_-based materials can be found at such scales, e.g., the size of rhombohedral magnesium calcite aggregates can be in the range 10–50 nm,^30^ whereas the average size of the cubic calcite nanoparticles has been reported at 62 nm.^31^ The low size of the rod-like crystals, i.e., aragonite, suggests that this material is highly reactive owing to its high surface area. To probe the internal structure, the selected area electron diffraction (SAED) pattern was also obtained. The low-magnification TEM image and the corresponding SAED diffraction pattern of a representative crystal of the analyzed sample is shown in Figure 4d. Based on the obtained results, the nucleate of Ca^2+^ denotes the calcite crystallization with a rhombohedral morphology. Furthermore, the maps revealed the presence of O (Figure 4e) and Ca (Figure 4f) in the rod-like particles, hence denoting the formation of CaCO_3_. Same morphological characteristics were observed at different magnifications, hence suggesting the homogeneity and consistency of this material. These results are typical for CaCO_3_^34^ and are in agreement with the above-mentioned results of the other analytical techniques.

### 3.7 Insight into the Carbon Mineralization Process and Future Potential

The very large reduction in EC, Ca, and particularly pH, whose scale is logarithmic, is a result of the dissolution of CO_2_ in CPW and the formation of carbonic acid, a weak acid that can be dissociated into hydrogen (H^+^) and bicarbonate (HCO_3_^–^) (or carbonate, CO_3_^2–^). This then reacts with the Ca content of CPW, which can be traced back to the dissolution of Ca(OH)_2_, and its precipitation as CaCO_3_. Depending on the effluent’s carbonate chemistry, additional CO_2_ could also be stored as (bi)carbonate. For example, it might also be possible to manipulate the CPW carbonate chemistry to hinder bicarbonate precipitation by using a salt or even adding sulfate and/or P (e.g., intentionally leaving some P in CPW). This can further improve the CO_2_ drawdown potential of this mineralization technology, particularly if the product water is released to the ocean where bicarbonate can remain safely stored for up to hundreds of thousands of years.^10,35^

For the sustainable scaling up of this carbonation process, the effect of typical operating parameters, such as CO_2_ concentration, flow rate,^36^ bubbling system,^37^ pressure,^38^ as well as the use of waste CO_2_ streams with different compositions and heat loadings, notably flue gases,^20,39^ should be considered. Engineering restrictions should be considered as well. For example, relatively pure and highly concentrated CO_2_ streams, such as the output of DAC systems that is intended for geological storage, would have a similar performance to the pure CO_2_ employed herein and ensure a fast carbonation reaction and relatively pure CaCO_3_ formation and precipitation. Nonetheless, the reacted and less concentrated CO_2_ stream should be captured and preferably recycled in the process, necessitating the need for a closed engineered reactor. In this case, it would also be possible to use a pressurized reactor, since high partial pressure of carbon dioxide (pCO_2_) shift the carbonate equilibrium and promote CaCO_3_ precipitation, while the CaCO_3_ particle size is also influenced by pressure.^38^ Carbonation efficiency could be further improved using CO_2_ microbubbles instead of bubbles generated with a conventional generator.^36^ The reactor geometry, CO_2_ flow rate, temperature, and pH should also be tailored to specific CO_2_-containing streams since these affect CaCO_3_ crystallization, whereas, if a specific polymorph of CaCO_3_ is the target, then the temperature can be controlled (e.g., temperatures >40 °C favor vaterite over calcite formation).^40^

Less concentrated CO_2_ streams, such as flue gases with typical CO_2_ concentrations ranging from as low as 3% (gas turbine) to as high as 33% (cement production),^41^ could also be used. In this case, depending on different parameters such as the flue gas composition and the depth of the CPW column, the capturing of the reacted flue gas might not be necessary since this might have been decontaminated, at least to a large extent. Nevertheless, the synthesized CaCO_3_ might also contain other impurities that were initially embedded in the flue gas matrix such as sulfur (SO_*x*_) and nitrogen oxides (NO_*x*_)^41^ or heavy metals such as arsenic (As),^42^ which can hinder its valorization. In these cases, CaCO_3_ will again be formed, since the Gibb’s free energy for CaCO_3_ formation suggests that the CO_2_ carbonation in Ca-rich wastewaters is relatively spontaneous.^43^ This is also the case for gypsum,^44^ suggesting that when flue gases with high sulfur content are used, then gypsum will also form and likely coprecipitate along with CaCO_3_. Furthermore, impurities contained in the flue gas can negatively influence the growth rate and nucleation of CaCO_3_, but, at the same time, could improve agglomeration.^39^ The bubbled effluent could also be affected by other contaminants such as As, hindering its release to the environment without further treatment.^42^ However, when CO_2_—air mixtures are concerned (e.g., DAC outputs), then this does not affect CaCO_3_ formation. For example, when 7.5 and 15% CO_2_—air mixtures were bubbled through a Ca(OH)_2_ solution, fine calcite particles were obtained in both cases.^45^

Flue gases also give off high amounts of heat and this could be beneficial for carbonation, since in direct mineral carbonation, elevated heat and temperature conditions can accelerate the carbonation reaction.^46^ The temperature also affect the morphology and size of the precipitants, with the morphology shifting away from calcite as the temperature increases.^37^ For example, temperatures >40 °C and the presence of magnesium ions favor the formation of needle-like aragonite metastable particles.^40^ Therefore, if flue gas from oxyfuel combustion is used, such as from oxyfuel limestone calcination which contains around 95% CO_2_,^21^ then aragonite will most likely form and precipitate rather than calcite, while gypsum and other minerals will have only a small contribution on the composition of the precipitant. If NO_*x*_ removal is desirable, then the denitrification of the flue gas should first be achieved, since the effectiveness of NO_*x*_ reduction is higher at the initial elevated temperatures of the flue gas.^47^ It should also be noted that when the carbonation of flue gas is achieved in Ca-rich wastewater such as CPW, then emission reductions, and not removals, would be typically achieved, unless, for example, the flue gas originates from a bioenergy with carbon capture and storage (BECCS) system.

Finally, atmospheric air could be used for bubbling, but the main issue of concern is its low CO_2_ content, roughly 0.04% or 400 ppm, which implies that long bubbling durations will be required to achieve high carbonation yields. Nonetheless, this might not be a limiting factor per se. For example, when the carbonation of a different Ca-rich effluent was examined, i.e., stabilized human urine, even though increasing the CO_2_ concentration from ambient (air) to 1% greatly increased the carbonation reaction (from 20.5 to 2.5 h), air bubbling was more cost-efficient.^48^ Air bubbling can also allow for the direct scaling up of this CDR approach. Specifically, in conventional wastewater treatment, aeration is an important step, whereby air is typically bubbled and evenly distributed across the wastewater matrix through bubble diffusers to promote microbial growth. With minor amendments, this infrastructure could be used for the direct scaling up of this CDR approach at industrial scale.

This is of major importance, given that P recovery from wastewater is on the rise. Specifically, each year, around 380 billion m^3^ of wastewater is produced and this is expected to increase by 24 and 51% in 2030 and 2050, respectively.^49^ These vast wastewater quantities have great potential for P recovery, since it has been estimated that MWW (human origin) alone contains 3.7 Mt yr^–1^ of P, of which 4% is currently technologically and economically recoverable.^12^ As the P-recovery technology matures and the regulations for P releases to receiving environments become even stricter, this number will increase, as will the volume of P-depleted alkalinized wastewater, typically effluents from struvite or calcium phosphate synthesis. Closing nutrient loops and the returning of P to the food production industry is a perquisite for circular economy,^50^ while P recovery from MWW can reduce reliance to phosphate rock extraction, whose reserves are finite and dwindling,^51^ and at the same time can credit the system with avoided impacts through fertilizer substitution.^52^ It also protects waterbodies from eutrophication,^53^ since P discharges from MWW is among the major causes of eutrophication.^54^

Even though calcium phosphate has comparable properties with phosphate rock and can be used for phosphoric acid production,^17^ its recovery from P-containing wastewaters has been mainly examined at lab and pilot scales.^55^ However, this is not the case for struvite recovery, where a strong and expanding industry exists. Full-scale struvite recovery systems already operate at industrial scale, with over 80 struvite production plants in operation worldwide, of which 24 are located in the EU producing up to 1250 t P from MWW and agro-industrial wastewater.^56^ Therefore, large volumes of P-depleted alkaline wastewater could be used for piloting and scaling up this CDR approach. It should be noted that in the case of struvite, magnesium (Mg) is used for struvite crystallization and precipitation, thus also producing highly alkaline wastewater. Similarly with CPW, this P-depleted wastewater could also be used for CO_2_ carbonation, and in this case, magnesium carbonate (MgCO_3_) will be produced.^43,57^ Colocation of DAC plants with existing struvite recovery plants can provide a stable and high-concentration CO_2_ stream that can catalyze the carbonation reaction in these effluents, or if aeration tanks are already in place, air could be used.

This CO_2_ mineralization process can also be part of a treatment train, whereby P is recovered, atmospheric CO_2_ is removed, CaCO_3_ is produced, and water is reclaimed. The synthesized CaCO_3_ could be used to produce zero-carbon lime, which is required for P recovery from wastewater; thus, a partial DAC system could be introduced. Not only this, but it appears that the quality of the bubbled effluent has also improved. As such, it might be even possible to discard the air-bubbled effluent directly to the environment, while water reclamation might become more feasible. The results are also indicative of other alkaline wastewater matrices, such as effluents from legacy iron and steel wastes (steel slags),^58^ which can be used for CaCO_3_ synthesis through interaction with CO_2_. For context, in the UK alone, over 190 million tonnes of legacy iron and steel slag are found,^59^ naturally producing large amounts of Ca-rich alkaline effluents which volumes can be greatly improved and used for CDR.

## Supporting Information Available

Schematic illustration of the proposed treatment system, XRD, FTIR, and XRF results (PDF)
