# Supplementary material for: Wastewater Treatment for Carbon Dioxide Removal
Source: ACS Omega. 2023 Oct 19;8(43):40251–9. doi: 10.1021/acsomega.3c04231 (PMC10620921; doi:10.1021/acsomega.3c04231)
Supplement: Supplementary file 1 — ao3c04231_si_001.pdf [file ao3c04231_si_001.pdf]

Supporting information

Wastewater treatment for carbon dioxide removal

Vhahangwele Masindi[1&2], Spyros Foteinis[3*], Phil Renforth[3], Efthalia Chatzisymeon[4]

[1]Magalies Water, Scientific Services, Research & Development Division, Erf 3475, Stoffberg street, Brits, 0250, South Africa

[2]Department of Environmental Sciences, College of Agriculture and Environmental Sciences, University of South Africa (UNISA), P. O. Box 392, Florida, 1710, South Africa

[3]Research Centre for Carbon Solutions, School of Engineering and Physical Sciences, Heriot-Watt University, Edinburgh EH14 4AS, United Kingdom

[4]School of Engineering, Institute for Infrastructure and Environment, University of Edinburgh, Edinburgh EH9 3JL, United Kingdom

[*] Corresponding Author: s.foteinis@hw.ac.uk

Contents

Figure S1: Schematic illustration of the overall treatment system S3

Figure S2: XRD results S4

Figure S3: FTIR results S5

Table S1: XRF results S6

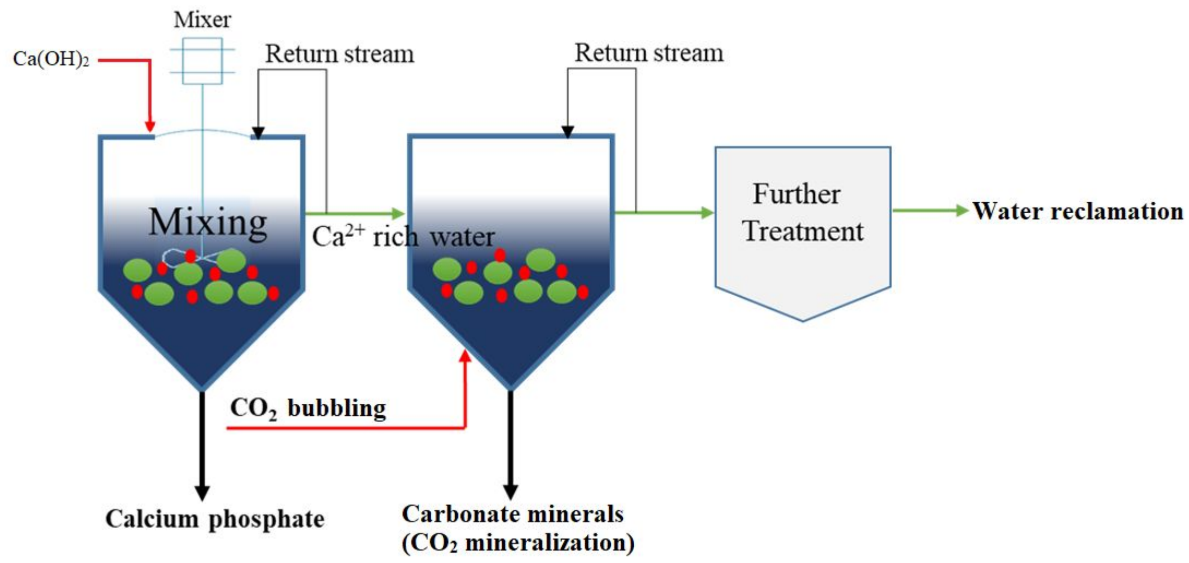

Figure S1: A schematic illustration of the overall treatment system.

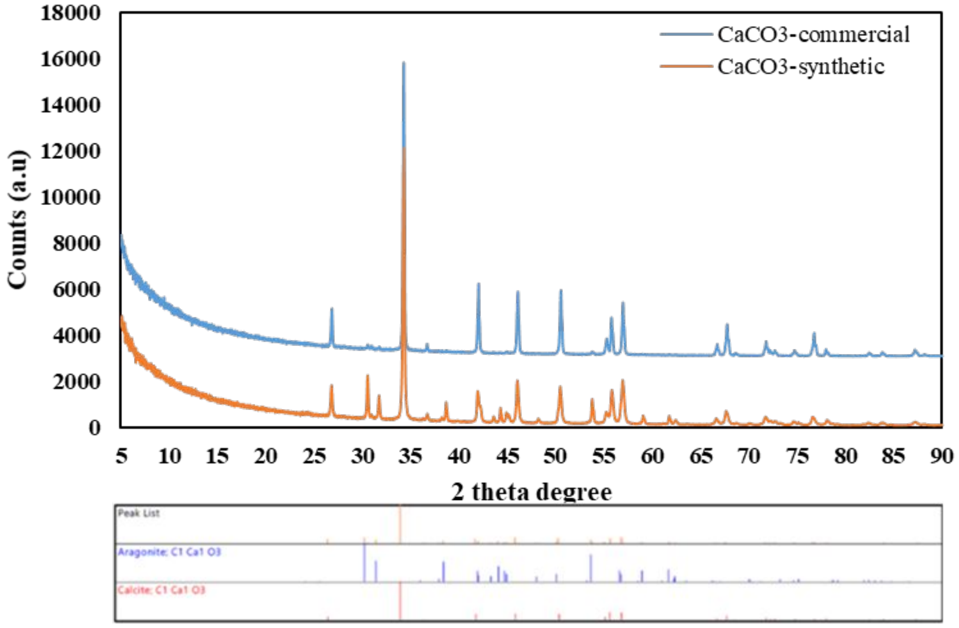

Figure S2: The XRD patterns and mineralogical compositions of the synthesized and commercially available $CaCO_3$.

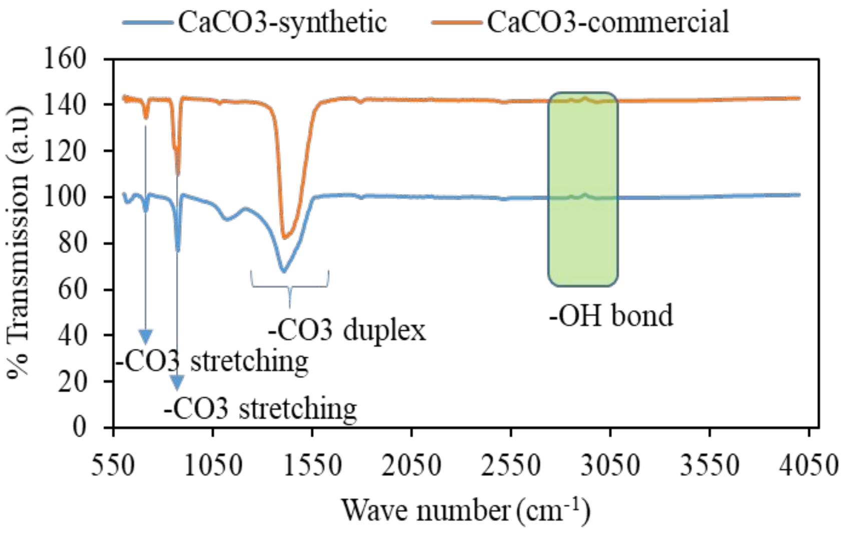

Figure S3: The metal functional groups of the synthesized and commercially available $CaCO_3$, as measured by FTIR.

Table S1: Elemental compositions of the synthesized and commercially available calcium carbonate as measured using XRF.

Element	Synthesized $CaCO_3$ (%)	Commercial $CaCO_3$ (%)
**CaO**	98.45	94.75
**Na_2O**	0.43	0.23
**SrO**	0.28	0.01
**SO_4**	0.15	0.03
**MgO**	0.10	0.52
**Cl**	0.08	0.01
**PO_4**	0.05	0.03
**K_2O**	0.05	0.01
**Th**	0.03	0.01
**Al_2O_3**	0.03	0.03
**Ru**	0.02	0.01
**Nb**	0.02	0.01
